# EDITORS INSIGHTS ON PUBLISHING OPPORTUNITIES FOR THE GLOBAL GERONTOLOGICAL COMMUNITY

**DOI:** 10.1093/geroni/igac059.303

**Published:** 2022-12-20

**Authors:** Edward Miller, Elizabeth Simpson

## Abstract

Global aging has proceeded at an unprecedented and accelerating rate. The aging of the population creates both opportunities and challenges for older adults, their families, and society in general. Importantly, there is substantial variation in the effects of and response to global aging both within and across nations depending, in part, on prevailing cultural expectations and values, political and economic imperatives, and social and demographic characteristics. Thus, while some regions and countries have responded with innovative policies and programs to better enable the growing cohort of older adults to remain active and engaged in the community, other regions and countries have struggled with their response or barely begun to plan for the rising population of older adults. This symposium assembles editors at five leading gerontology journals to demonstrate the role that peer-reviewed scholarship can play in disseminating knowledge that informs gerontological research, policy, and practice internationally. Editors include: Edward Alan Miller, PhD, FGSA, Journal of Aging & Social Policy; Jeffrey Burr, PhD, FGSA, Research on Aging; Julie Robison, PhD, FGSA,, Journal of Applied Gerontology; Sandra Torres, PhD, FGSA, Ageing & Society; and Julie Hicks Patrick, PhD, FGSA, International Journal of Aging & Human Development. Each presenter will review the scope, content, and focus of their journals and the role and opportunities for international scholarship.

